# Wildlife forensic examination in Russia: the practice and perspectives

**DOI:** 10.1080/20961790.2016.1243082

**Published:** 2016-12-16

**Authors:** Svetlana A. Smirnova, Georgy G. Omelyanyuk, Victoriya V. Gulevskaya, Shamil N. Khaziev

**Affiliations:** a The Russian Federal Centre of Forensic Science of the Ministry of Justice of the Russian Federation, Moscow, Russia; b Peoples' Friendship University of Russia (RUDN University), Moscow, Russia; c Institute of State and Law, Russian Academy of Sciences, Moscow, Russia

**Keywords:** Forensic science, hair analysis, animal fur, wildlife forensic, Eurasian wildlife forensic networks

## Abstract

Two case studies are described which show application of forensic expertise to establish important circumstances related to the investigation of the crimes against wildlife flora and fauna. Forensic study of the animal hair is a method for investigation of the crimes against wildlife objects which is used more frequently during the recent years. The perspectives of development of the new branch of forensic research in Russia are formulated, and the proposals and recommendations for developing the Eurasian wildlife flora and fauna network are formulated in this article.

## Introduction

The frequency of the wildlife crime, dynamic development of the Customs Union and the necessity of the customs procedures unification including legal expertise support for the customs authorities in relation to the wild flora and fauna objects, urge the forensic expertise development in Russia.

The wildlife forensic in Russia advocates the need for forensic support for the wildlife crime investigations: domestic, foreign and international practices and scope for improvement. The presentation examines the key developments in the provision of forensic support for investigations of the wildlife crime, and advocates the need to incorporate the wildlife forensics as a separate branch of forensic inquiry in the Russian Federal Center of Forensic Science (RFCFS) of the Russian Ministry of Justice with cooperation of the Severtsov A.N. Institute of Ecology and Evolution of the Russian Academy of Sciences (Institute of ecology and evolution).

The Open Expert Group named Eurasian Wildlife Forensic Networks (EAWFN) was created in 2014 to enhance efficiency of the forensic examination in this sphere. EAWFN was formed by the Forensic Institutions of the Republic of Kazakhstan, the Kyrgyz Republic, the Russian Federation, the Republic of Tajikistan, in order to promote disclosure of crimes against the wildlife [1–3].

Recently, the forensic support for investigation of crimes against the representatives of the family *Felidae* has received special attention. This attention is by the necessity to preserve relatively sparse wild population of these species threatened by the increasing illegal hunting and smuggling. In particular, the illegal trade of the skins of jungle cat (*Felis chaus*), leopard (*Panthera pardus*), lynx (*Lynx lynx*), irbis (*Uncia uncia*), derivatives of Amur (Siberian) tiger (*Panthera tigris altaica*) are quite common. In this paper, we describe some examples of expert practice of RFCFS when the research objects were animals or their part and derivatives, belonging to the family *Felidae*.

## Materials and methods

The materials of this study are the objects of the wildlife fauna, belonging to the family *Felidae*, their parts and derivatives. The following methods were applied: macro-morphological and microscopic observations, organoleptic investigation.

Fifty animal skins were delivered for expertise to RFCFS; nine reference samples from the collection of the Research Zoological Museum of the Biological Faculty, Moscow State Lomonosov University (further referred as Zoological Museum). In the framework of this expertise, hair from all 50 samples were studied as well as the hair from the collections of Zoological Museum: wildcat (*Felis silvestris lybica*, three samples; *Felis silvestris*, three samples), jungle cat (*Felischaus*) (three samples). The latter reference samples were studied first.

## Results

**Figure 1.**
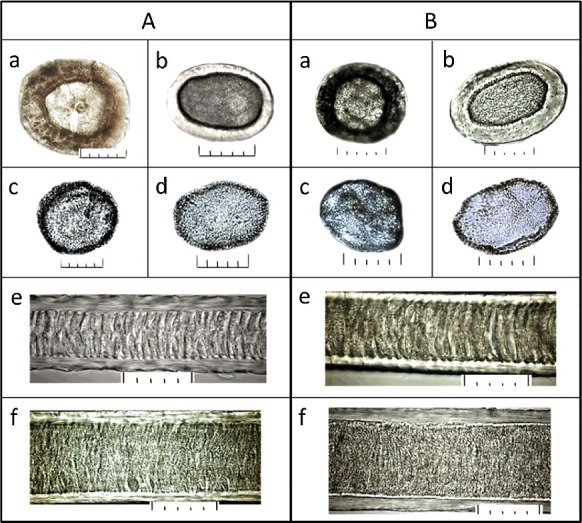
Detailed macro- and micro-morphological comparison of hair specimens: (A) microstructure of the hair of *Felis silvestris* from collection; (a)(b) cross sections in the most expanded part of a hair (granna) at a pigmented pile hair and an achromous pile hair, respectively; (c)(d) medulla disks in the same area at a pigmented pile hair and an achromous pile hair, respectively; (e)(f) structure of a medulla in the base of a pile hair and a granna of a pile hair, respectively. (B) microstructure of a typical hair from objects ##1–50; (a)(b) cross sections in the most expanded part of a hair (granna) at a pigmented pile hair and an achromous pile hair, respectively; (c)(d) medulla disks in the same area at a pigmented pile hair and an achromous pile hair, respectively; (e)(f) structure of a medulla in the base and the granna of a pile hair, respectively. The scale at all photos: one small division on a large-scale ruler is equal to 10 μ (microns), respectively 5 divisions = 50 μ (microns).

The detailed macro- and micro-morphological comparison of the guard hairs and down of the samples 1–50 with the samples from Zoological Museum has shown that they have characteristics typical for the species of wildcat (*Felissilvestris*) (Figure 1).

Further conclusions about chemical or technological treatment of the studied objects were done on the basis of organoleptic study. It was demonstrated that the skins passed through certain technological processes, most probably there were processes in small workshops for commercial purposes.

**Figure 2.**
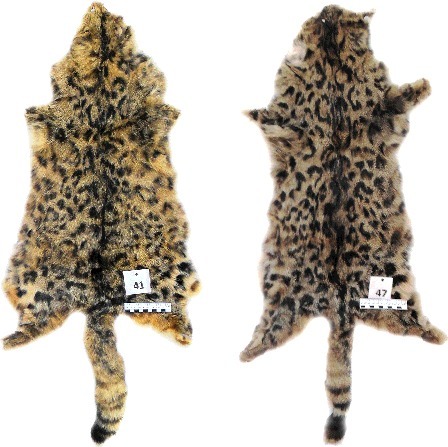
Artificial colouration of the skins created using stencils (objects #41 and #47).
- The species *Felis silvestris* is spread in Europe, western Asia and northern Africa. Within Russia, its habitat includes southern parts of Dagestan, Chechnya, Stavlopol and rasnodar districts, Kabardino-Balkaria, Northern Ossetia, Adygea.

The following conclusions were made based on the research results:

- 50 skins submitted for expertise are the derivatives of wildlife objects and belong to the animals of the species *Felis silvestris*, the mammal of the order Carnivora, family Felidae, genus *Felis*.
- The species *Felis silvestris* is included in the Appendix II of the Convention on International Trade in Endangered Species (CITES) of Wild Fauna and Flora.
- All analysed skins are passed through technological treatment (Figure 2).

**Figure 3.**
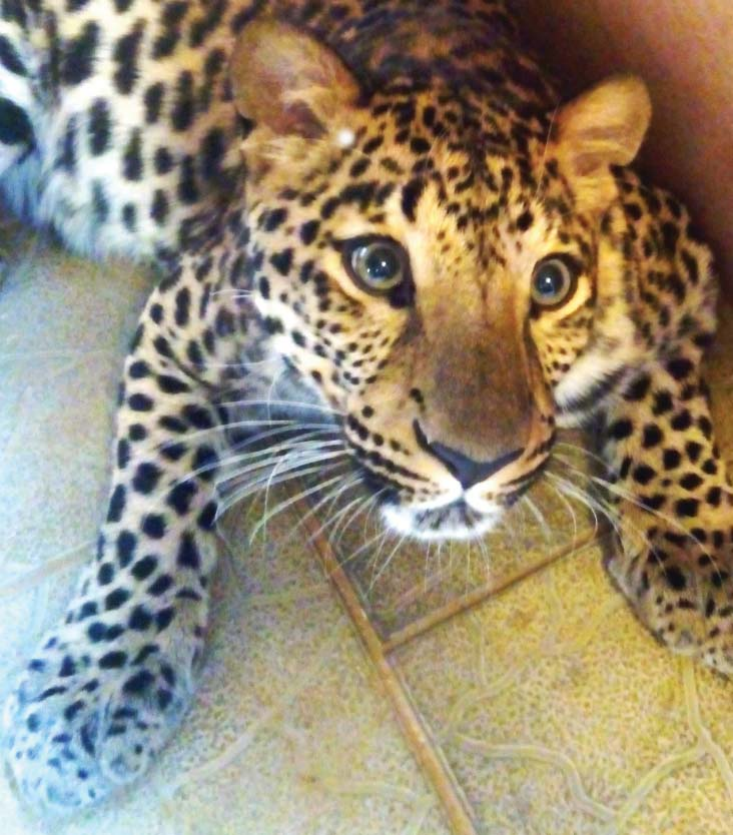
General appearance of the object of study.

One more expertise case:

In the RFCFS, a forensic inquiry of a wildlife fauna object was conducted based on the investigation of the photographic images and the specimens of the hair collected at the scene of crime. Figure 3 shows the external appearance of the object.

The comparative study of the submitted objects was carried out using hair specimens of different subspecies and hybrids of the leopard species from the collection of the RFCFS obtained due to the exchange programme in the framework of the EAWFN.

**Figure 4.**
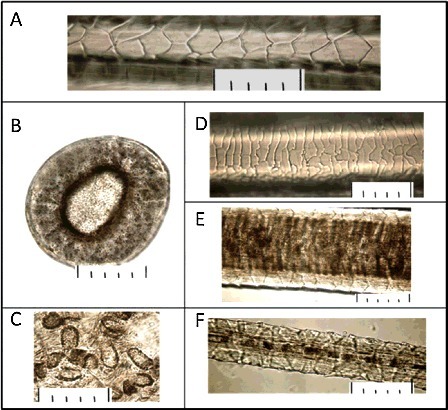
Microstructure of leopard hair collected at the crime scene: (A) cuticle in the base of a pile hair; (B) cross section of a pile hair in a grana; (C) the medulla disks received in the course of alkaline thermohydrolysis; (D) cuticle in an expanded part of a pile hair; (E) medulla in an expanded part of a pile hair; (F) microstructure of a fur hair. The scale at all photos: one small division on a large-scale ruler is equal to 10 μ (microns), respectively 5 divisions = 50 μ (microns).

**Figure 5.**
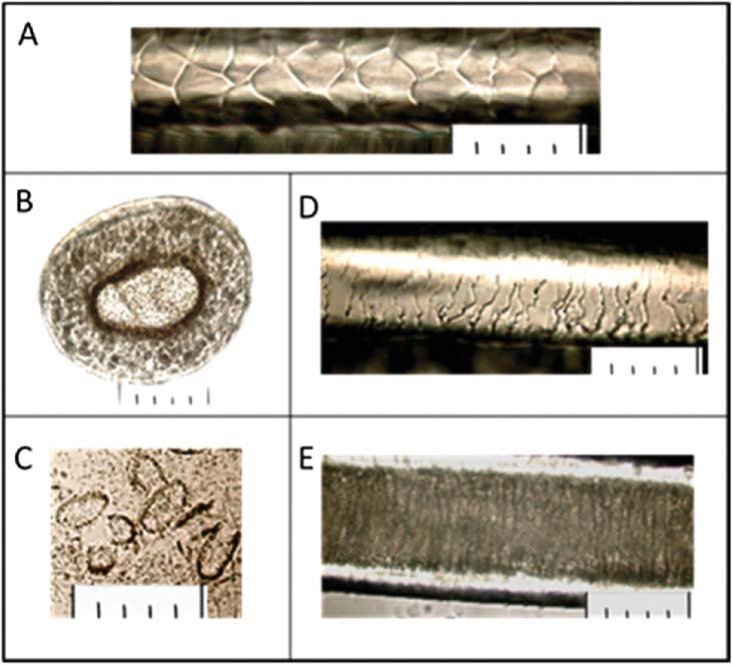
Microstructure of leopard hair (a hybrid of *Panthera pardus orientalis* and *Panthera pardus ciscaucasicus*) from the collection: (A) cuticle in the base of a pile hair; (B) cross section in an expanded part of a pile hair; (C) the medulla disks received in the course of alkaline thermohydrolysis; (D) cuticle in an expanded part of a pile hair; (E) medulla in an expanded part of a pile hair. The scale at all photos: one small division on a large-scale ruler is equal to 10 μ (microns), respectively 5 divisions = 50 μ (microns).

The detailed comparative study of the hair from the scene of crime and from the referential collections has shown that the compared hair has the structure similar to that of the hair of the hybrid type of the animal (hybrid of the Far East and Near East subspecies). The compared characteristics of the hair are presented in Figures 4 and 5.

The forensic investigation has demonstrated that the animal, obtained during the visual study of the crime scene belongs to the Ieopard species (*Pantherapardus*), being a hybrid of two subspecies: Far Eastern (*Panthera pardus orientalis*) and Near Eastern (*Panthera pardus ciscaucasicus*). The purity versus hybridity of the leopard from the crime scene was confirmed by the molecular genetic analysis conducted in the Institute of Ecology and Evolution.

The leopard species (*Pantherapardus*) is included in the Appendix I of the CITES, as well as in the International Red Book and the Red Book of Russian Federation.

According to the publication [4], the hybrid animals, in this case the hybrid of two leopard subspecies (*Panthera pardus orientalis* and *Panthera pardus ciscaucasicus*), do not conform to the genetic status of the animals suitable for re-introduction (population restoration). The natural hybridization of these two subspecies is improbable, taking into account the large distance between their habitats. This means that this animal most probably was bred in the captivity.

## Discussion

Summarizing the experience of the forensic expertise for the inquiries on illegal hunting of the animals belonging to the family *Felidae* clearly demonstrates the necessity for detailed study of their biological characteristics, formation of traces of these animals, development of the methods of collection and study of substantial evidences. Recently, complexes of forensic expertise have been applied for the inquiries about the crimes against the wildlife objects: zoological, traseological, ballistic, veterinary and some other special types of expertise. Very interesting seems to be the study of the specific features of the stripe pigmentation pattern of the skins of some species of the family *Felidae*, for example Amur tiger (*Panthera tigris altaica*).

As mentioned earlier, wide possibilities for improvement of the forensic expertise of the wildlife representatives of the family *Felidae* are generated through the development of molecular genetic research methods. DNA investigation of the wild cats could allow not only detection of the species, but in some cases even identification of individual [5–7].

Russian forensic experts are interested in the cooperation with the foreign colleague to exchange experience of the forensic expertise practices, new methods, methodologies, technical means and reference materials. Regular direct personal contacts between the experts and scientists are apparently the most efficient and promising link. This is most important for the experts from neighbouring countries, inhabited by the same species of the family *Felidae*. As for RFCFS, it initiated a programme for integrating the research experience of the wildlife flora and fauna by the forensic institutions as well as by the research teams of the Russian Academy of Sciences and other scientific organizations and universities.

## Conclusion

In the perspective of combating the crime in the sphere of the wildlife plants and animals utilization, the priority should be given to the synthesis of the experience in carrying out forensic expertise of the wildlife flora and fauna objects, scientific methodological support of this branch of the forensic inquiry, international consolidation and coordination of scientific and methodological policy in relation to the wildlife flora and fauna objects by means of the EAWFN development.
